# Supplementary material for: Chinese Consumers’ Attitudes and Potential Acceptance toward Artificial Meat
Source: Foods. 2021 Feb 7;10(2):353. doi: 10.3390/foods10020353 (PMC7915407; doi:10.3390/foods10020353)
Supplement: Supplementary file 1 [file foods-10-00353-s001.pdf]

Survey about artificial meat

Survey about Artificial Meat

* Required

Introduction and context of the survey

Artificial meat, also known as in vitro meat, cultured meat, lab meat, clean meat and also synthetic meat, is a novel food produced in laboratories using animal muscle stem cells, but does not come directly from a living animal and which proliferate in culture. The production of artificial meat is the subject of media enthusiasm to feed the growing human population. In order to solve the increasing concerns about environment (global warming) and ethic problems (animal welfare, animal suffering and
slaughtering) but also the weaknesses of the conventional meat production (limited farming resources and ever increasing population), scientific research is devoted to introduce and develop on a large scale artificial meat as a new meat product in the future.

This study is conducted by French researchers from INRAE (Theix, 63), ISARA (Lyon, 69) and Bordeaux Sciences-Agro (Bordeaux, 33). The purpose of this survey is to study the opinion of consumers about this biotechnology and to investigate their preferences and/or their aversions. That is why you are being asked to participate in this research project by taking an online survey, which should not take more than 15 minutes of your time. In accordance with the code of ethics of survey organizations, be assured that your personal data and the information you will provide will be kept confidential and not used except within average results for the statistical objectives of the survey. You should be 18 years old at least to complete this survey, but you do not need any specific skills nor academic degrees. Clicking on the next button to access the questionnaire implies your consent to participate. However, you can stop answering the survey anytime if you like. In this case, the information you have provided will not be saved. Only the display at the end of the questionnaire after page 8 implies the archiving of information. For any questions, you can write to 'futurecellmeat@gmail.com'

Principles of artificial meat production

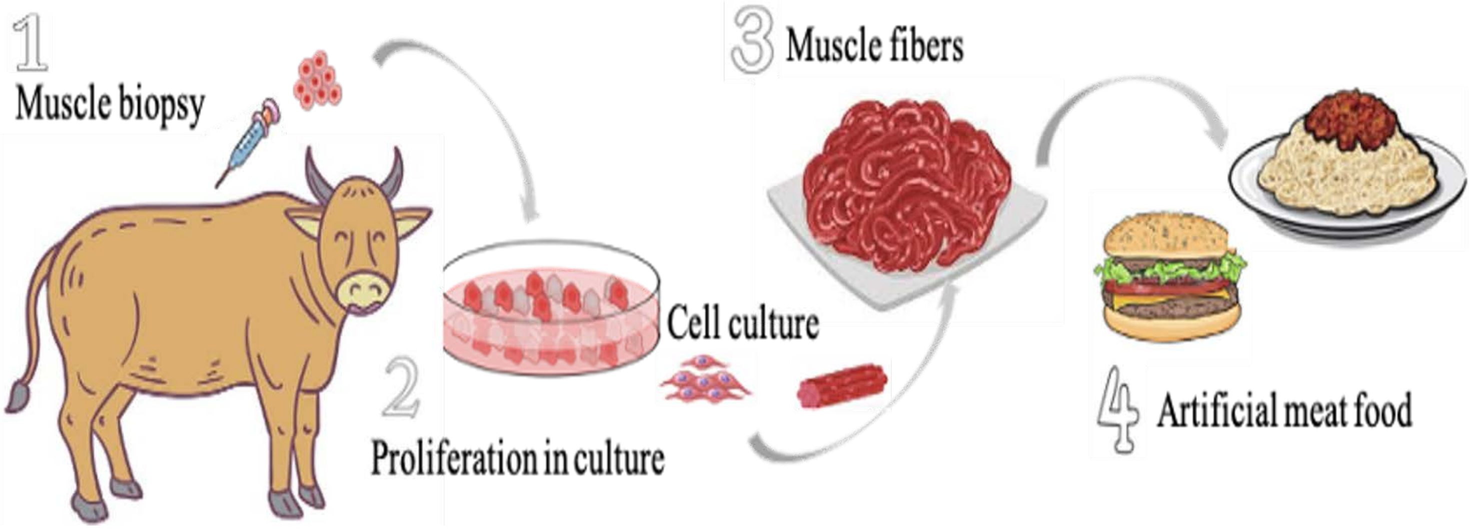

Demographic information

1. Sex *

Please tick only one box.

- Female
- Male
- Does not wish to answer

2. Continent or country of origin *

Please tick only one box.

- France
- Other countries within the European Union
- European countries outside the European Union
- China
- Russia
- Middle East and Asia excluding China and Asian Russia
- North America
- South America
- Africa
- Oceania
- UK

3. Age range *

Please tick only one box.

- 18-30 years of age
- 31-50 years of age
- 51 years of age and more

4. Education *

Please tick only one box.

- [ ] Primary school & High school
- [ ] College
- [ ] Master
- [ ] PhD (thesis)
- [ ] Does not wish to answer
- [ ] Other: ____________

5. Activity area *

Please tick only one box.

- [ ] Scientist within the meat sector
- [ ] Scientist outside the meat sector
- [ ] Not scientist but within the meat sector
- [ ] Not scientist and outside the meat sector

6. Monthly net income *

Please tick only one box.

- <1 k yuan (155 USD)
- 1 k – 3 k yuan (155 – 464 USD)
- 3 k – 5 k yuan (464 – 773 USD)
- 5 k – 8 k yuan (773 – 1236 USD)
- 8 k – 10 k yuan (1236 – 1546 USD)
- >10 k yuan (1546 USD)

7. Meat consumption *

Please tick only one box.

- Never: vegetarian or vegan diet
- Rarely: weekly or less
- Regularly: several times a week
- Daily or within each meal

Preamble

8. Have you ever heard about artificial meat before? *

Please tick only one box.

- ( ) Yes
- ( ) No

9. What are the most important criteria during your food shopping? *

Check all that apply.

- [ ] Ethics (breeding systems, production methods, animal welfare...)
- [ ] Environmental impacts
- [ ] Price
- [ ] Sensorial quality (taste, juiciness, tenderness...)
- [ ] Aspect / Appearance
- [ ] Energy intake (calories)
- [ ] Nutritional value (proteins, vitamins...)
- [ ] Origin and traceability
- [ ] Safety
- [ ] Marks and/or labels

Other: [ ] ______________________

Societal challenges

10. Do you think the on-farm breeding and meat industry cause important ethical problems (e.g. animal suffering, slaughtering...) ? *

Please tick only one box.

	1	2	3	4	5	
Much less / I disagree	⬭	⬭	⬭	⬭	⬭	Much more / I agree

11. Do you think that the on-farm breeding and meat industry cause important environmental problems (e.g.huge water consumption, greenhouse gas emissions ...) ? *

Please tick only one box.

	1	2	3	4	5	
Much less / I disagree	⬭	⬭	⬭	⬭	⬭	Much more / I agree

12. To deal with these potential problems, do you think that reducing our meat consumption could be a good solution? *

Please tick only one box.

	1	2	3	4	5	
Much less / I disagree	⬭	⬭	⬭	⬭	⬭	Much more / I agree

13. How ethical do you think artificial meat would be compared to conventional meat (e.g. able to significantly improve animal welfare, reduce animal suffering ...)? *

Please tick only one box.

	1	2	3	4	5	
Much less / I disagree	⬭	⬭	⬭	⬭	⬭	Much more / I agree

14. How eco-friendly do you think artificial meat would be compared to conventional meat (e.g. able to significantly reduce the environmental footprint of farming such as water consumption, global warming effect, greenhouse gas emissions ...)? *

Please tick only one box.

	1	2	3	4	5	
Much less / I disagree	⬭	⬭	⬭	⬭	⬭	Much more / I agree

15. Do you think artificial meat would have negative impacts on traditional livestock farming and meat industry (e.g. employment...)? *

Please tick only one box.

	1	2	3	4	5	
Much less / I disagree	⬭	⬭	⬭	⬭	⬭	Much more / I agree

16. Do you think artificial meat would have negative impacts on territories and rural life (e.g. biodiversity, tourism, landscape maintenance, vitality of the territories...)? *

Please tick only one box.

	1	2	3	4	5	
Much less / I disagree	⬭	⬭	⬭	⬭	⬭	Much more / I agree

Characteristics of the product

17. How healthy, safe and with a high-nutritional-value do you think artificial meat would be compared to conventional meat (i.e. in terms of proteins, vitamins...)? *

Please tick only one box.

	1	2	3	4	5	
Much less	⬭	⬭	⬭	⬭	⬭	Much more

18. How tasty do you think artificial meat would be compared to conventional meat (i.e. similar to conventional meat in terms of taste)? *

Please tick only one box.

	1	2	3	4	5	
Much less	⬭	⬭	⬭	⬭	⬭	Much more

Potential interests

19. Would you accept artificial meat as a viable alternative compared to conventional meat in the future, compared toother meat substitutes (such as soy proteins) or other solutions (such as reducing food waste or developing our farming practices)? *

Please tick only one box.

- ( ) Yes and I already eat meat substitutes/alternatives
- ( ) Yes but I don't eat meat substitutes/alternatives
- ( ) No but I eat meat substitutes/alternatives
- ( ) No and I don't eat meat substitutes/alternatives

20. What could be the reasons for you to try artificial meat? (multiple choice) *

Check all that apply.

- [ ] Solution to feed the ever-growing human population
- [ ] Attractive price compared to conventional meat
- [ ] Ethic (improve animal welfare and reduce animal slaughtering)
- [ ] Less risk of zoonosis (disease that can be transmitted from animals to people)
- [ ] Attractivity of high-tech technologies
- [ ] Clean product
- [ ] Eco-friendly product
- [ ] Curiosity
- [ ] I am not willing to try this product

Other: [ ] ____________________

21. Why would you NOT be willing to try artificial meat? (multiple choice) *

Check all that apply.

- [ ] Unnatural
- [ ] Less tasty/appealing
- [ ] Worries about safety
- [ ] More expensive than conventional meat
- [ ] Feeling of reluctance (disgust, nervousness ...)
- [ ] Negative impact on local farmers (jobs ...)
- [ ] Negative impact on territories and rural life (biodiversity, tourism, landscape maintenance, vitality of territories ...)
- [ ] Less trust in laboratories and artificial meat start-ups
- [ ] Significant environmental footprints

Other: [ ] ____________________

22. What would you expect from artificial meat? (multiple choice) *

Check all that apply.

- [ ] Adequate nutrition
- [ ] Tasty/Taste similar to conventional meat
- [ ] Safety
- [ ] Less expensive than conventional meat
- [ ] Less environmental footprints
- [ ] Reduction of farming
- [ ] No farming
- [ ] No animal pain/suffering
- [ ] Nothing

Other: [ ] ____________________

Perception

23. What do you think of artificial meat? *

Please tick only one box.

- It is promising and/or acceptable
- It is fun and/or intriguing
- It is absurd and/or disgusting

24. Do you have emotional resistance (e.g. disgust, nervousness) to try artificial meat? *

Please tick only one box.

	1	2	3	4	5	
Much less						Much more

25. Would you be willing to try artificial meat? *

Please tick only one box.

- Definitely yes
- Probably yes
- Unsure
- Probably not
- Definitely not

26. In which context(s), would you be willing to eat artificial meat regularly? (multiple choice) *

Check all that apply.

- [ ] At the restaurant
- [ ] At home
- [ ] In ready-to-eat meals: lasagna, burger...
- [ ] I do not want to eat artificial meat regularly

Other: [ ] ____________________

27. How much would you be willing to pay for artificial meat compared to conventional meat? *

Please tick only one box.

- ( ) Much less than conventional meat, even nothing at all
- ( ) Less than conventional meat
- ( ) Same price as conventional meat
- ( ) More than conventional meat
- ( ) Much more than conventional meat

28. Do you think artificial meat is realistic? *

Please tick only one box.

- On the short term: from 1 to 5 years
- On the medium term: from 6 to 15 years
- On the long term: more than 16 years
- Never

Development strategies

29. To which extent do you think that the private research model (by start-ups) is relevant for potentially developing research on artificial meat? *

Please tick only one box.

	1	2	3	4	5	
Much less						Much more

30. To which extent do you think that scientific public research must invest (time and funding) to develop this biotechnology? *

Please tick only one box.

	1	2	3	4	5	
Much less						Much more

This content is neither created nor endorsed by Google.

Google Forms
